# Challenges of Implementing the PRISM 2.0 Trial for Supporting Social Connectivity Through Technology

**DOI:** 10.1093/geroni/igab046.1190

**Published:** 2021-12-17

**Authors:** Walter Boot, Sara Czaja, Dana Plude

## Abstract

Following the success of the Personal Reminder Information and Social Management (PRISM) trial, which found that a specially designed computer system for older adults can enhance social connectivity and reduce loneliness among older adults at risk for social isolation, the PRISM 2.0 trial sought to replicate and extend these results to a new technology platform (tablet rather than desktop) with expanded social features and diverse populations of older adults, including older adults living in rural areas, assisted living communities, and senior housing. This symposium discusses the aims of the trial conducted by the Center for Research and Education on Aging and Technology Enhancement (CREATE), challenges encountered (including challenges related to the COVID-19 pandemic), and solutions to those challenges. S. Czaja will begin with an overview of the PRISM 2.0 system and the trial. J. Sharit will discuss challenges encountered working within the context of assisted living facilities and with impaired participants. This will be followed by a discussion of technical challenges encountered during the course of the trial presented by N. Charness. W. Rogers will present training issues involved (both with respect to participants and assessors). Finally, W. Boot will describe challenges encountered with measuring and quantifying technology use during the trial. Lessons learned are applicable to many types of technology interventions administered in diverse contexts. D. Plude, Deputy Director in the Division of Behavioral and Social Research of NIA, will serve as discussant.

